# 17-AAG Kills Intracellular *Leishmania amazonensis* while Reducing Inflammatory Responses in Infected Macrophages

**DOI:** 10.1371/journal.pone.0049496

**Published:** 2012-11-13

**Authors:** Antonio Luis de Oliveira Almeida Petersen, Carlos Eduardo Sampaio Guedes, Carolina Leite Versoza, José Geraldo Bomfim Lima, Luiz Antônio Rodrigues de Freitas, Valéria Matos Borges, Patrícia Sampaio Tavares Veras

**Affiliations:** 1 Laboratório de Patologia e Biointervenção, Fundação Oswaldo Cruz-BA, Salvador, Bahia, Brazil; 2 Laboratório Integrado de Microbiologia e Imunoregulação, Fundação Oswaldo Cruz-BA, Salvador, Bahia, Brazil; 3 Departamento de Anatomia Patológica e Medicina Legal, Universidade Federal da Bahia, Salvador, Bahia, Brazil; University of California Merced, United States of America

## Abstract

**Background:**

Leishmaniasis is a neglected endemic disease with a broad spectrum of clinical manifestations. Pentavalent antimonials have been the treatment of choice for the past 70 years and, due to the emergence of resistant cases, the efficacy of these drugs has come under scrutiny. Second-line drugs are less efficacious, cause a range of side effects and can be costly. The formulation of new generations of drugs, especially in developing countries, has become mandatory.

**Methodology/Principal Findings:**

We investigated the anti-leishmanial effect of 17-(allylamino)-17-demethoxygeldanamycin (17-AAG), an HSP90 inhibitor, *in vitro*. This inhibitor is currently in clinical trials for cancer treatment; however, its effects against intracellular *Leishmania* remain untested. Macrophages infected with *L. amazonensis* were treated with 17-AAG (25–500 nM) and parasite load was quantified using optical microscopy. Parasite load declined in 17-AAG-treated macrophages in a dose- and time-dependent manner. Intracellular parasite death became irreversible after 4 h of treatment with 17-AAG, and occurred independent of nitric oxide (NO) and superoxide (O_2_^−^) production. Additionally, intracellular parasite viability was severely reduced after 48 h of treatment. Interestingly, treatment with 17-AAG reduced pro-inflammatory mediator production, including TNF-α, IL-6 and MCP-1, yet IL-12 remained unaffected. Electron microscopy revealed morphological alterations, such as double-membrane vacuoles and myelin figures at 24 and 48 h after 17-AAG treatment.

**Conclusions/Significance:**

The HSP90 inhibitor, 17-AAG, possesses high potency under low dosage and reduces both pro-inflammatory and oxidative molecule production. Therefore, further studies are warranted to investigate this inhibitor’s potential in the development of new generations of anti-leishmanials.

## Introduction

The leishmaniases are a complex of vector-borne diseases affecting 12 million people worldwide, caused by protozoans of the genus *Leishmania,* which are transmitted by the bite of infected sand flies. This complex of diseases manifests in two main clinical forms: visceral leishmaniasis, which can be fatal if left untreated, and tegumentary leishmaniasis, whose mucocutaneous and cutaneous forms induce lesions that are generally self-healing, but may leave scars or deformities. The worldwide prevalence and severity of leishmaniasis has led the World Health Organization (WHO) to consider it as one of the most serious infectious diseases [1]. Due to the complexity of host-parasite interaction, as well as contradictory results from vaccination models, the control of infection is mainly dependent on chemotherapeutic intervention [2].

A variety of treatment options currently exist. Pentavalent antimonials, such as Pentostam and Glucantime, have been used to treat leishmaniasis for the last 70 years [3], [4]. However, decreasing effectiveness due to increased resistance [5] has limited their application. The antimicrobials Pentamidine and Amphotericin B represent the second line of treatment. Pentamidine has demonstrated efficacy against cutaneous leishmaniasis, however, its side effects include cardiotoxicity, renal failure and the development of diabetes at high dosage [6], [7]. Liposomal Amphotericin B is the treatment of choice in the USA and Europe, as it has been proven effective against leishmaniasis with minimal side effects. However, its high cost limits the applicability for many patients in developing countries [8]. The antiprotozoal miltefosine has demonstrated efficacy against cutaneous leishmaniasis and visceral leishmaniasis in oral administration; however, teratogenic effects restrict its widespread usage. In addition, this is a long-term use drug associated with treatment discontinuation and, as a consequence, development of resistance [4], [9]. While the antibiotic paromomycin offers the same cure rate as amphotericin B, its poor oral absorption has led to the development of parenteral and topical formulations. However, local pain, ototoxicity and nephrotoxicity are the most frequently reported adverse events associated with this drug [4], [9]. Taken together, the inadequacies of these current treatment options implicate the urgent development of new drugs to cure leishmaniasis with greater efficacy, minimal side effects and low cost.

Heat shock protein 90 (HSP90) is highly abundant in mammalian cells and known to be induced during stress responses. This protein is an ATP-dependent chaperone known to be involved in the stabilization, correct folding, and assembly of several client proteins, including kinases, transcription factors and proteins involved in cell-cycle control [10], [11], [12]. Some of these client proteins are known to be key oncogenic proteins that are upregulated in cancer cells [11], [13], [14]. Protozoan parasites also express HSP90 [15], which is known to play a crucial role in the stabilization of heat-labile proteins within these cells [16], [17]. The modulation of HSP90 by specific inhibitors or by heat shock stress provokes profound modifications in parasite differentiation processes [18]. The use of Geldanamycin (GA), an HSP90-specific inhibitor, to treat erythrocytes infected with *Plasmodium* protozoa arrested intracellular parasite growth, blocked progression of protozoa from the ring stage to the trophozoite stage and resulted in the death of intra-erythrocyte parasites [19], [20], [21]. A previous study demonstrated HSP-90 inhibition using a GA analog, 17-(allylamino)-17-demethoxygeldanamycin (17-AAG), which reduced *Plasmodium* sp growth in an *in vivo* mouse model of malaria [21]. These authors also demonstrated that 17-AAG inhibited *Trypanosoma evansi* growth *in vitro* and enhanced infected animal survival rates from 0% in the untreated control group to 60% in the 17-AAG-treated group [21]. In addition, the protozoan *Toxoplasma gondii,* when treated with GA, resulted in reduced parasite entry and intracellular growth in host cells [22]. In sum, these data suggest that HSP90 participates in parasite entry and influences survival in host cells [21], [22].

In *Leishmania* spp., HSP90 represents 2.8% of the total cell protein content [23]. This protein plays a role in the differentiation process of the parasite from promastigote to amastigote form [18]. Indeed, inhibition of parasite HSP90 by heat shock stress or treatment with specific inhibitors, such as GA or radicicol (RD), induces the arrest of promastigote growth and the transformation of promastigotes into rounded amastigote-like forms, together with size reduction and flagellum loss [18]. Furthermore, the inhibition of HSP90 using GA was able to promote apoptosis-induced parasite death, when the promastigotes were incubated at 37°C pH 5.5, environmental conditions similar to those found in intracellular *Leishmania*-induced parasitophorous vacuoles [24]. Taken together, these data support the notion that HSP90 is a potential target for chemotherapeutic intervention for the control of parasitic diseases [18], [25].

The less-toxic GA analog, 17-AAG, is currently in phase II clinical trials for the treatment of several cancers. This analog HSP-90 inhibitor shares many common features with GA [26], however 17-AAG binds to the HSP90 ATP pocket with greater affinity than GA, more efficiently impairing ATP hydrolysis, and, consequently, HSP90 chaperone activity. This effect ultimately promotes the proteasomal cleavage of HSP-90 client proteins more efficiently [27]. This study represents the first attempt to evaluate the effect of 17-AAG on *Leishmania amazonensis*-infected macrophages. The data presented herein demonstrate that when macrophages were treated with nanomolar concentrations of 17-AAG, the clearance of parasite infection *in vitro* was obtained.

## Materials and Methods

### Ethics Statement

The Animal Care Facility at CPqGM/FIOCRUZ provided male and female CBA and C57BL/6 mice. The animals were housed under specific pathogen-free conditions, fed commercially available rations and given water *ad libitum*. CBA and C57BL/6 mice were euthanized at 6 to 12 weeks of age. The animal husbandry and housing conditions, as well as the experimentation protocols at our facility, comply with the International Guiding Principles for Biomedical Research Involving Animals and have been approved by the CPqGM Institutional Review Board for Animal Experimentation.

### Reagents

17-AAG was purchased from InvivoGen (San Diego, California, EUA). Dimethyl sulfoxide (DMSO) (SIGMA, St Louis, MO, USA) was used to prepare a 5 mM stock solution of 17-AAG, stored in aliquots at −20°C, until use. This solution was further diluted in cell culture medium to the desired concentrations at time of use. Other reagents used were: Schneider’s insect medium (SIGMA), gentamycin (SIGMA), sodium bicarbonate (SIGMA), 4-(2-hydroxyethyl)-1-piperazineethanesulfonic acid (HEPES) (SIGMA), glutamine (SIGMA), lipopolysaccharide LPS (SIGMA), lucigenin (SIGMA), wortmannin (SIGMA), AlamarBlue® (Invitrogen, Carlsbad, CA, USA), amphotericin B (Fungizone, GIBCO, Carlsbad, CA, USA), fetal calf serum (FCS) (GIBCO), DMEM medium (GIBCO), heparin (ARISTON, Brazil), ciprofloxacin (CLARIS, India) and IFN-γ (R&D systems, Minneapolis, MN, USA).

### Parasite Cultures

To avoid a loss of parasite infectivity, the *L. amazonensis* (MHOM/BR/87/BA125), *Leishmania major* (MHOM/RI/−/WR173) and *Leishmania infantum* (MCAN/BR/89/BA262) strains were maintained by serial passages in C57BL/6 mice. Following parasite isolation from the popliteal lymph nodes of infected mice, axenic parasites were maintained by serial passages in Schneider’s insect medium supplemented with 10% FCS and 50 µg/mL of gentamycin. Axenic cultures were maintained until a maximum of seven passages.

### Inhibiting Effect on Axenic Promastigotes

Axenic promastigotes of *L. amazonensis*, *L. major* or *L. infantum* at a concentration of 5×10^6^ parasites/mL, cultivated in Schneider's complete medium, were treated with differing concentrations of the HSP90-inhibitor, 17-AAG (25, 125, 300 or 500 nM). The anti-leishmanial amphotericin B was used as positive control at 270 nM. At 48 h after treatment, the effect of these drugs on parasite growth was evaluated by directly counting live motile parasites using a Neubauer chamber.

### Macrophage Cultures, Infection and Treatment

Briefly, thyoglycollate-elicited macrophages were obtained from the peritoneal cavities of CBA mice and cultivated according to modified protocols established by Gomes *et al*. (2003) [28]. All cells were recovered in heparinized saline (20 UI/mL) and centrifuged at 300 × g for 10 min at 4°C. Next, macrophage cultures were maintained at 2×10^5^ cells/mL in DMEM complete medium (DMEM medium supplemented with 10% inactivated FCS, 2 g/L of sodium bicarbonate, 25 mM HEPES, 1 mM of glutamine and 0.2% of ciprofloxacin). Cells were cultivated at 37°C in 5% CO_2_ for 4–16 h before infection. Next, the antileishmanial activity of 17-AAG against intracellular *L. amazonensis* parasites was assessed in elicited peritoneal macrophages infected with stationary-phase promastigotes (10∶1 ratio). After 6 h of incubation with parasites, all macrophage groups were washed and submitted to a variety of treatment protocols: a) macrophages were treated with either 25, 125 or 500 nM of 17-AAG for an additional 6, 24 or 48 h to evaluate drug effects at early time after infection. Positive control cultures were treated with amphotericin B at a variety of concentrations: 250, 500, 1,000, 2,000, and 4,000 nM for 48 h; b) macrophages were incubated for 48 h, then treated with 25, 125 or 500 nM of 17-AAG for an additional 48 h to evaluate drug effects at later stages following infection; c) macrophages were treated with 500 nM of 17-AAG for 2, 4, 8, 12 or 24 h. At the end of each treatment period, all cells were washed and subsequently reincubated in 17-AAG-free medium to obtain a total incubation time of 72 h regardless of treatment period. After 6 h of infection, the control group was incubated in complete DMEM medium containing DMSO at the same concentration used in 17-AAG-treated cultures for 24 h, then washed and incubated for an additional 48 h (total incubation time = 72 h). After 72 h, all macrophage groups were washed and stained with hematoxylin/eosin (H&E) at the end of experimentation procedures. Parasite load was assessed using optical microscopy by quantifying both the percentage of infected cells and the number of parasites per infected cell. For each experimental condition, at least 400 cells were counted per coverslip in triplicate to sextuplicate.

### Assessment of Macrophage Viability

Macrophage cultures were maintained at 2×10^5^ cells/mL in DMEM complete medium and treated with 125, 500, 1,000, 3,000, 5,000 and 10,000 nM of 17-AAG or its diluent for 48 h. Next, cultures were washed twice and the medium was replaced by DMEM complete medium containing 10% AlamarBlue®. Cells were reincubated for 4 h at 37°C, in 5% CO_2_. Next, reagent absorbance was measured at the wavelengths of 570 nm and 600 nm using a spectrophotometer (SPECTRA Max 340 PC). Ethanol-fixed cells were used as positive controls.

### Assessment of Parasite Intracellular Viability

First, macrophage cultures were infected for 6 h and either wortmannin for 24 or 48 h, or left untreated. At the end of these incubation times, all cultures were washed and the culture medium was replaced with 1 mL of fresh Schneider’s complete medium and the remaining intracellular parasites were incubated at 23°C. At the end of five days, the intracellular amastigotes that had transformed into motile extracellular promastigotes were quantified by determining the number of viable parasites using a Neubauer chamber [29].

### Quantification of Superoxide Production by Macrophages

Drug effect on superoxide (O_2_^−^) production was assessed using: i) a chemiluminescence (CL) assay to monitor O_2_^−^ production during phagocytosis, and ii) a fluorescence assay using an O_2_^−^ specific hydroethidine probe to determine intracellular O_2_^−^ production. Elicited peritoneal macrophages were plated at 10^6^ parasites in 2 mL of complete DMEM medium. For both assays, macrophages were stimulated with LPS at 1 µg/mL in the presence or absence of 17-AAG at 500 nM, or with 500 nM of 17-AAG alone, for 20 h. O_2_^−^ production by peritoneal inflammatory macrophages was measured during 20 min after the addition of *L. amazonensis* promastigotes at a 10∶1 ratio at 37°C, using a lucigenin (25 µM) chemiluminescence (CL) method in a photon-counting device comprising a gallium arsenide photomultiplier tube (Hamamatsu R943) [30]. Chemiluminescence emissions from sample dishes, incubated at 37°C in a sealed chamber, were reflected and focused onto the photomultiplier tube. The emitted signal was fed directly to a frequency counter unit, and data were collected in units of photon counts per second [30]. For the fluorescent probe assay, hydroethidine at 5 µM was added according to manufacturer protocols (Invitrogen). Cell fluorescence was measured (FACSort; BD Biosciences) and expressed as mean fluorescence intensity (MFI) using a flow cytometer. In both assays, unstimulated macrophages infected with *L. amazonensis* promastigotes were used as negative controls.

### Quantification of Cytokine and Nitric Oxide (NO) Production by Macrophages

Elicited peritoneal macrophages were plated at 10^6^ cells/mL of complete DMEM medium. Macrophages were stimulated with IFN-γ (100 UI/mL) for 20 h. Next, macrophages were infected with *L. amazonensis* stationary phase promastigotes for 6 h. Then, macrophage cultures were washed to remove all non-internalized parasites, the DMEM cell medium was replaced and IFN-γ stimulation was reapplied together with 500 nM of 17-AAG. These cultures were incubated and cell supernatants were collected at 24 h or 48 h to determine mediator levels or NO production, respectively. Concentration of released mediators was determined using an inflammatory CBAKit (BD Biosciences, San Jose, CA, USA) in accordance with manufacturer protocols. NO production was assessed in cell supernatants by determining nitrite accumulation, using the Griess method [31].

### Transmission Electron Microscopy

Macrophages were first infected with *L. amazonensis* for 6 h and treated with 500 nM of 17-AAG for 6, 12, 24 or 48 h. Control macrophages were similarly infected with *L. amazonensis,* but left untreated. Cells were then fixed in a solution containing 2.5% glutaraldehyde grade II, 2% formaldehyde and 2.5 mM CaCl_2_ in 0.1 M sodium cacodylate buffer adjusted to pH 7.2. Next, cells were post-fixed in the same buffer with 1% osmium tetroxide and 0.8% potassium ferricyanide, then dehydrated in an acetone series and embedded in Polybed resin. Thin sections were taken and stained with uranyl acetate and lead citrate. Observations were performed using a Zeiss 109 or Jeol 1230 transmission electron microscope.

### Statistical and Data Analyses

Data are shown as an average of 3–6 independent experiments (mean ± SEM) performed in at least triplicate, or a representative experiment from a series of independent experiments performed in sextuplicate (mean ± SD). The number of experiments performed is indicated in each figure legend. Using GraphPad 5.0 software, normality testing (Kolmogorov-Smirnov) determined the use of a parametric method (Student’s *t*-test, one-way ANOVA with Dunnett’s Multiple Comparison Test and post-test for linear trend) or non-parametric method (Mann-Whitney test). Results were considered statistically significant if *p*<0.05.

The selective index (SI), based on a nonlinear regression analysis was calculated as follows: SI =  LC_50_/IC_50,_ where LC_50_ corresponds to the lethal concentration able to reduce macrophage viability by 50% and the IC_50_ value represents the inhibitory concentration needed to kill 50% of intracellular parasites [32].

## Results

### 17-AAG Reduced Axenic Growth of *Leishmania* and Controlled Parasite Infection

**Figure 1:**
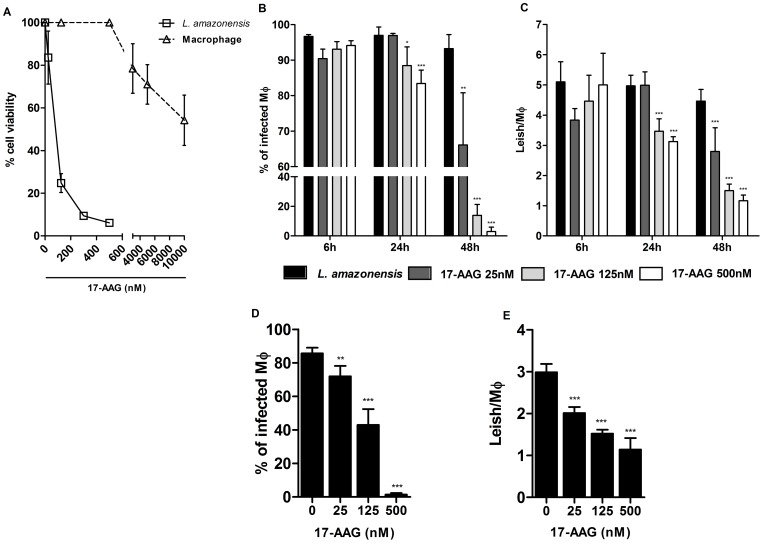
Inhibition of axenic growth of *Leishmania* and reduction in parasite load by 17-AAG. (**A**) Axenic promastigotes were exposed to several concentrations of 17-AAG (25, 125, 500 nM) for 48 h and the number of viable parasites was assessed as described in Materials and Methods. Data are presented as the percentage inhibition of parasite growth related to untreated controls (4,754×10^7^). Bars represent means ± SD of one representative out of two experiments performed in sextuplicate (one-way ANOVA, Dunnett’s Multiple Comparison Test, ***p<0.0001, post-test for linear trend, *p*<0.0001). (**B, C**) Drug effects at early times after infection. Following 6 h of incubation with parasites, macrophage cultures were treated for 6, 24 and 48 h with specific concentrations of 17-AAG (25, 125, 500 nM); (**D, E**) Drug effects at later stages after infection. Following 6 h of incubation with parasites, macrophage cultures were reincubated for additional 48 h then submitted to treatment with specific concentrations of 17-AAG (25, 125, 500 nM). Bars represent means ± SD of one representative experiment out of two performed in sextuplicate (one-way ANOVA, Dunnett’s Multiple Comparison Test, *p<0.05, **p<0.001, ***p<0.0001, post-test for linear trend, *p*<0.0001).

In order to evaluate whether 17-AAG had a direct effect on parasite axenic growth, quantification under microscopy was performed. Amphotericin B was used as positive control against *Leishmania* parasites. The effect of both drugs on parasite growth was quantified at 48 h after treatment and the control exhibited complete inhibition at a concentration of 270 nM. All parasite groups treated with 17-AAG showed significantly reduced axenic growth, and a statistically significant lower number of parasites was measured following 48 h of treatment in comparison to untreated control promastigotes. Treatment with 25 nM of 17-AAG reduced parasite growth by 21.22±4.6% in relation to the control group (*p*<0.0001) (Fig. 1A). Concentrations of 125, 300 and 500 nM reduced parasite growth by 74.74±4.3, 90.57±1.5 and 93.89±1.4%, respectively (*p*<0.0001, Fig. 1A). The IC_50_ value calculated for *L. amazonensis* in axenic culture was 64.56±7 nM. 17-AAG was also able to inhibit the growth of other *Leishmania* species: *L. major* promastigotes at very low concentrations, with an IC_50_ value of 79.6±3.7 nM, and *L. infantum* promastigotes at low concentrations, with an IC_50_ value of 169.1±18.3 nM. These findings indicate that 17-AAG induced a static effect on promastigote axenic growth.

Next, the anti-leishmanial activity of 17-AAG against intracellular *L. amazonensis* parasites was evaluated by determining parasite load at time points representative of early stages of infection: 6, 24 and 48 h. Untreated cells were used as controls, and the percentage of infected cells ranged from 93.2±3.9% to 96.9±2.3% throughout all incubation periods (Fig. 1B). After 6 h of treatment, no statistically significant differences were observed in the percentage of infected cells between the 17-AAG-treated groups and control cells. However, after 24 h of treatment, the percentage of infected cells treated with 125 and 500 nM of 17-AAG fell significantly to 88.4±5.2% (*p*<0.05) and 83.4±3.8%, respectively, in comparison to 96.9±2.3% in the control group (*p*<0.0001, Fig. 1B). The percentage of infected cells decreased further in infected macrophages submitted to a 48-hour treatment incubation time, even at concentrations as low as 25 nM: to 66.1±14.6%, compared to 93.2±3.9% in untreated controls (*p*<0.0001, Fig. 1B). This reduction was much more pronounced in cells treated at concentrations of 125 nM and 500 nM to 13.9±7.3% and 3.0±2.9%, respectively (*p*<0.0001, Fig. 1B).

The number of parasites per untreated cell ranged from 4.4±0.3 to 5.0±0.6 (Fig. 1C). Similarly to what was observed in the percentage of infected cells, no significant differences were observed in the number of parasites when comparing the 17-AAG-treated groups to control cells after 6 h of treatment. However, after 24 h of treatment, a decrease in the number of parasites per macrophage was observed in cells treated with 125 and 500 nM of 17-AAG to 3.4±0.4 and 3.1±0.2, respectively ((*p*<0.0001, Fig. 1C) in comparison to control cells: 4.9±1.2. After 48 hours of treatment, a pronounced reduction in the number of parasites per macrophage was observed at concentrations of 25, 125, and 500 nM, to 2.7±0.7, 1.5±0.2, and 1.1±0.1, respectively, in comparison to controls: 4.5±0.3 (*p*<0.0001, Fig. 1C). Amphotericin B was used as a positive control for intracellular parasite death at concentrations of 250, 500, 1,000, 2,000, and 4,000 nM. Even the lowest concentration resulted in complete clearance of intracellular parasites.

The authors also evaluated the toxicity effect of 17-AAG on host macrophages. When treated with 125, 500, 1,000 and 3,000 nM of 17-AAG for 48 h, macrophage viability remained unaltered, as assessed by an AlamarBlue® assay (Fig. 1A). However, treatment with 5,000 nM of 17-AAG reduced host cell viability by 28.9±9.2% (*p*<0.05, Fig. 1A) and treatment with 10,000 nM reduced host cell viability by 45.7±11.7% (*p*<0.0001, Fig. 1A). The resulting LC_50_ value was calculated as 10,830±1,700 nM of 17-AAG, while the IC_50_ value was determined to be 149±7 nM. Hence, the corresponding SI value was calculated as follows: SI = 10,830 nM/149 nM = 72.68.

In order to evaluate the treatment’s effect at later stages of infection, 17-AAG was added to axenic cultures after 48 h of infection, the time needed for parasites to transform from promastigote to amastigote form [33]. After 48 h of treatment, all treated groups exhibited a decrease in parasite load. A significant reduction was observed in the percentage of infected cells in a dose-dependent manner: from 85.6±3.4% in controls, to 72.0±6.2% (*p*<0.005) at 25 nM; 42.9±9.4% (*p*<0.0001) at 125 nM; and 1.4±0.8% (*p*<0.0001) at 500 nM (Fig. 1D). A decrease was similarly observed when assessing the number of parasites per macrophage: from 3.0±0.1 in controls, to 2.0±0.1 (*p*<0.0001) at a concentration of 25 nM; to 1.5±0.1 (*p*<0.0001) at 125 nM; and to 1.1±0.2 (*p*<0.0001) at 500 nM (Fig. 1E). In sum, these data show that treatment with 17-AAG effectively reduced the percentage of *L. amazonensis*-infected macrophages, as well as the number of parasites per macrophage, in a time- and dose-dependent manner. Furthermore, this inhibitor was shown to successfully kill parasites that had already differentiated into intracellular amastigotes under infection conditions lasting as long as 96 hours.

### The Irreversible Effect of 17-AAG on *Leishmania* Parasites

**Figure 2:**
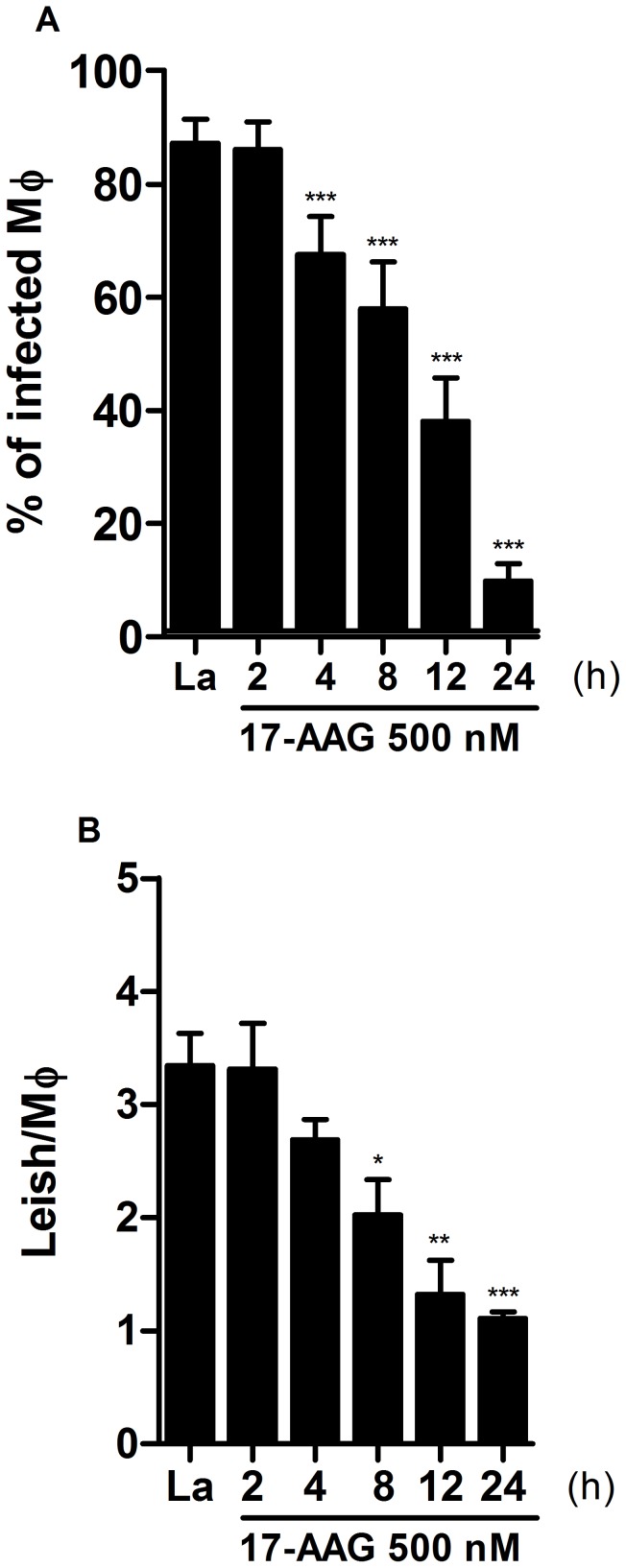
Irreversibility of treatment with 17-AAG on intracellular *Leishmania*. To assessment the reversibility of parasite growth inhibition by treatment with 17-AAG parasite load was determined by quantifying the percentage of infected macrophages (**A**) and the number of parasites per macrophage (**B**) as described in Materials and Methods. Bars represent means ± SD of one representative experiment out of two performed in sextuplicate (one-way ANOVA, ***p<0.0001, Dunnett’s Multiple Comparison Test, *p<0.05, **p<0.001, ***p<0.0001, post-test for linear trend, *p*<0.0001).

**Figure 3:**
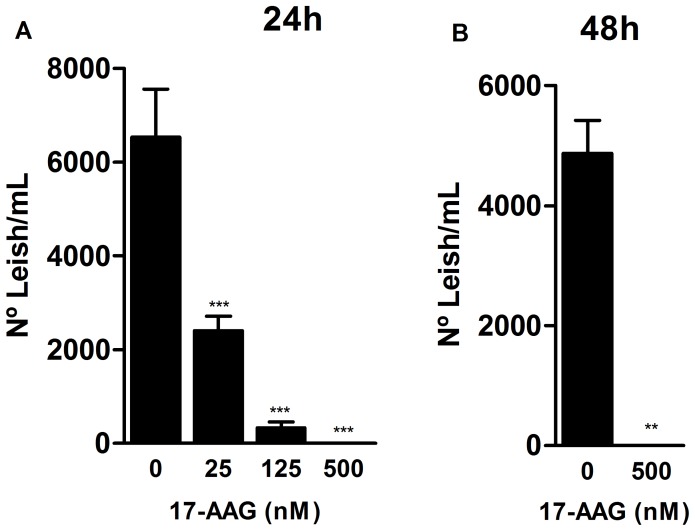
Reduction of parasite intracellular viability by 17-AAG. Treatment’s effect on parasite viability was assessed after 24 h (**A**) and 48 h (**B**) of infection, as described in Materials and Methods. Bars represent means ± SD of one representative experiment out of two performed in sextuplicate (one-way ANOVA, ***p<0.0001, Dunnett’s Multiple Comparison Test, *p<0.05, **p<0.001, ***p<0.0001, post-test for linear trend, *p*<0.0001; Mann Whitney test, **p<0.001).

Next, the reversibility of the inhibitor’s effect on parasite load was assessed. Infected macrophages were treated with 500 nM of 17-AAG for 2, 4, 8, 12 or 24 h. At the end of each treatment period, all cells were washed and subsequently reincubated in 17-AAG-free medium to obtain a total incubation time of 72 h, regardless of initial treatment period (Fig. 2). Control groups were incubated in 17-AAG-free medium for 24 h, then washed and incubated for an additional 48 h (total incubation time = 72 h). The cultures treated with 17-AAG for 2 h showed no reduction in either the percentage of infected cells, or in the number of parasites per infected macrophage (Fig. 2A–B). However, after 4 h of treatment an irreversible reduction in the percentage of infected cells was observed: from 87.2±4.2% in the control group to 67.5±6.7 (*p*<0.0001) (Fig. 2A). After 8 h of treatment, this irreversible effect was more pronounced in the percentage of infected cells: to 57.8±8.3 (*p*<0.0001), as well as in the number of parasites per macrophage: from 3.3±0.2 in the control group to 2.0±0.3 (*p*<0.05) (Fig. 3A–B). After 24 h of treatment, the percentage of infected cells fell dramatically to 9.7±3.1%, (*p*<0.0001) (Fig. 2A), as well as the number of parasites per infected cell to 1.1±0.06 (*p*<0.0001) (Fig. 2B). These data indicate that the inhibitory effect of 17-AAG on *Leishmania* parasites *in vitro* is irreversible at treatment times longer than 4 h.

### 17-AAG Reduced Intracellular Parasite Viability

Next, experiments were performed to evaluate whether 17-AAG was able to significantly reduce intracellular parasite viability. After 24 h of treatment, a significant reduction in the number of viable parasites was observed at concentrations of 25 nM: 2,408±306 parasites/mL, 125 nM: 329±127 parasites/mL, and 500 nM: 0.5±0.2 parasite/mL (*p*<0.0001), in comparison to the untreated control group: 6,535±1,024 parasites/mL (*p*<0.0001) (Fig. 3A). After 48 h, no viable parasites remained in the group treated with 500 nM (Fig. 3B). These findings demonstrate that the rate of parasite survival was considerably affected by treatment with 17-AAG in a time- and dose-dependent manner.

### 17-AAG Reduced the Oxidative Response of *Leishmania*-infected Macrophages

**Figure 4:**
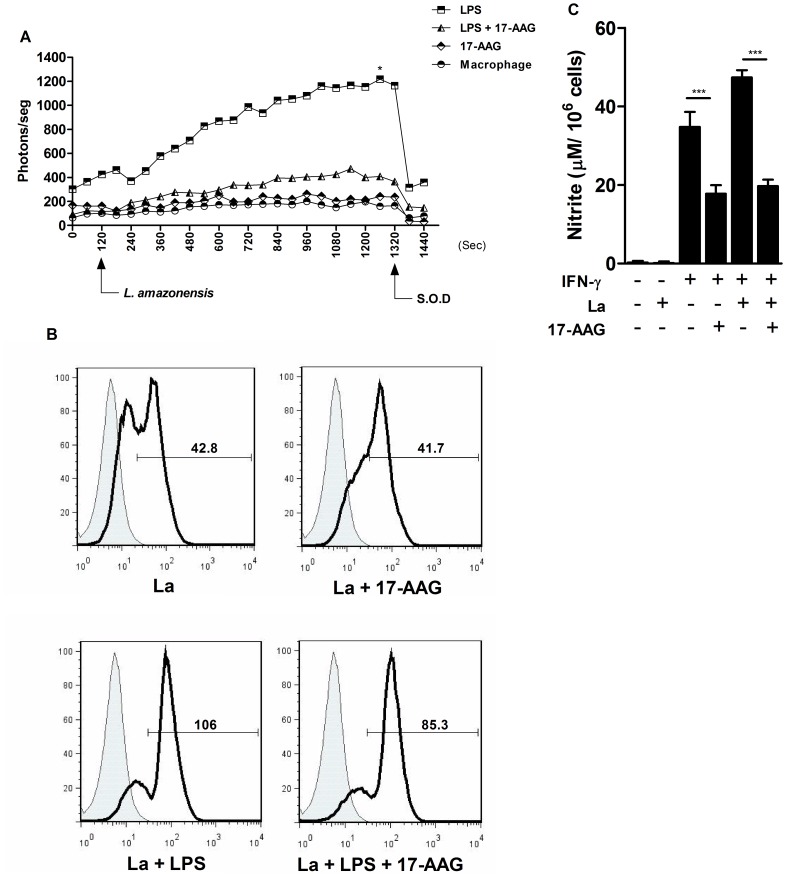
Reduced O_2_ ^−^ **and NO production in macrophage cultures treated with 17-AAG.** (**A**) O_2_^−^ production was measured at early stages of infection using a lucigenin-derived CL method. Points on the graph represent photon emissions per second by macrophage cultures 2 min prior to the addition of *L. amazonensis* promastigotes, as well as throughout the incubation period of 20 min. Data are derived from one representative experiment out of four performed in uniplicate (Mann Whitney test, p = 0.028); (**B**) Intracellular O_2_^−^ production was assessed by determining cell fluorescence in the presence of hydroethidine (5 µM) and expressed as MFI using a flow cytometer. The histogram overlay depicts the MFI of hydroethidine-labeled cells (solid lines) in comparison to unlabeled control cells (shaded areas). Data are derived from one representative experiment out of three performed in uniplicate (Mann Whitney test, *p* = 1). (**C**) NO production was measured by detecting nitrite in the supernatants of 17-AAG-treated cells, as described in Materials and Methods. Bars represent NO production measurement expressed as means ± SD of one representative experiment out of four performed in triplicate or more (Student’s *t* test, ***p<0.0001).

In order to investigate the mechanism by which 17-AAG induces intracellular *Leishmania* death, the authors measured the production of leishmanicidal molecules by macrophages, such as superoxide (O_2_^−^) and nitric oxide (NO). First, O_2_^−^ production was assessed using a CL assay. Figure 4A depicts O_2_^−^ production by stimulated macrophages following an infection time of 20 min. As expected, prior to infection, LPS-stimulated macrophages produced high levels of O_2_^−^, reaching a maximum value (Rmax) of 424 photons/sec. By contrast, macrophages treated with LPS and 500 nM of 17-AAG produced lower levels of O_2_^−^, reaching an Rmax of 116 photons/sec. When *L. amazonensis* promastigotes were added to LPS-stimulated cultures, a marked increase in O_2_^−^ production was observed, reaching an Rmax of 1,217 photons/sec (Fig. 4A), 2.6 times higher than the Rmax from LPS-stimulated cell cultures treated with 17-AAG (471 photons/sec, *p* = 0.02) (Fig. 4A). Macrophages treated with 17-AAG alone, as well as the untreated control cells, exhibited no alterations in O_2_^−^ production, with an Rmax value of 261 photons/sec in macrophages treated with 17-AAG alone, and 200 photons/sec in untreated macrophages (*p*>0.05, Fig. 4A). In order to confirm that photon release was due to O_2_^−^ production, the rapid decay values of photon emission, in response to the addition of S.O.D. (2.5 UI/mL) was verified at the end of the assay (Fig. 4A).

Intracellular O_2_^−^ production was then determined at later infection times using a fluorescent assay with hydroethidine, an O_2_^−^ specific fluorescent probe. Figure 4B depicts the mean fluorescence intensity (MFI) values emitted by macrophages, which are proportional to O_2_^−^ production by these cells. Unstimulated macrophages treated with 17-AAG (500 nM) for 24 h (MFI = 41.7) produced O_2_^−^ at similar levels to those produced by untreated control macrophages (MFI = 42.8) (Fig. 4B). However, when LPS-stimulated macrophages were treated with 17-AAG (500 nM), O_2_^−^ values fell from 106 to 85.3 MFI (Fig. 4B), a reduction that was not statistically significant.

Thereafter, the authors evaluated the effect of 17-AAG (500 nM) on NO production by detecting nitrite levels using the Griess reaction. Untreated control cells, both uninfected and infected with *L. amazonensis* promastigotes, produced NO at levels under the detection limit of the curve. Uninfected macrophages previously stimulated with IFN-γ released 34.7±3.8 µM of NO in culture medium. The addition of 17-AAG to these cell cultures reduced NO production to 17.7±2.2 µM (*p*<0.0001, Fig. 4C). Similarly, the addition of 17-AAG to infected cells previously stimulated with IFN-γ reduced NO production to levels significantly lower than those produced by infected macrophages previously stimulated with IFN-γ: from 47.3±1.8 µM to 19.7±1.6 µM (*p*<0.0001, Fig. 4C).

In sum, these findings suggest that O_2_^−^ and NO play no role in the induction of intracellular *Leishmania* death triggered by 17-AAG.

### 17-AAG Modulates Cytokine Production by Infected Macrophages

**Figure 5:**
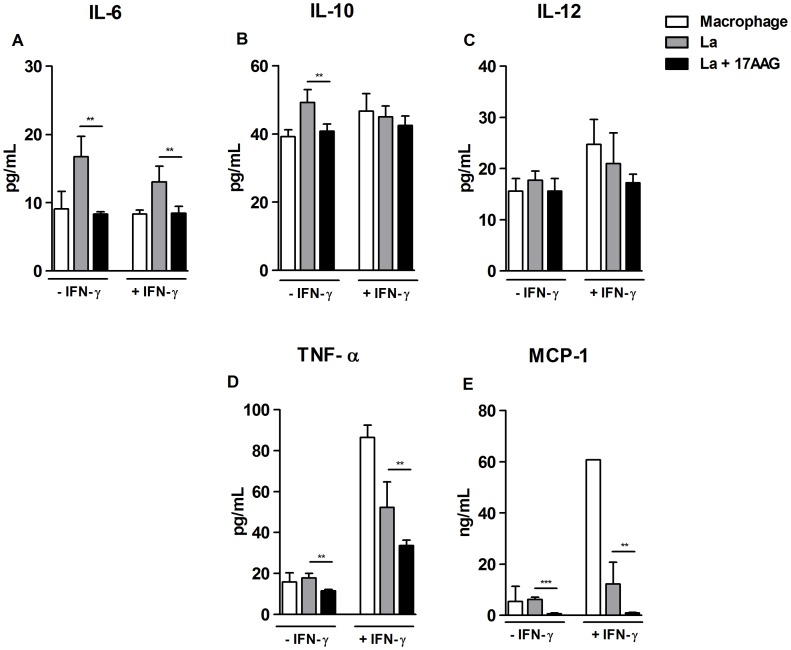
Modulation of mediator production by treatment with 17-AAG. Mediators released by 17-AAG-treated cells were measured in cell supernatants using an inflammatory CBAKit, as described in Materials and Methods: (**A**) IL-6; (**B**) IL-10; (**C**) IL-12; (**D**) TNF-α; (**E**) MCP-1. Bars represent means ± SD of a single experiment performed in sextuplicate (Student’s *t*-test and Mann-Whitney, **p<0.001, ***p<0.0001).

Next, the authors evaluated the effects of 17-AAG (500 nM) on the production of pro-inflammatory cytokines by infected macrophages. Negative control macrophages, both non-activated and uninfected, i.e. not previously stimulated with IFN-γ, secreted the following cytokines in culture supernatants: 8.3±0.5 pg/mL of IL-6; 39.1±2.0 pg/mL of IL-10; 15.5±2.4 pg/mL of IL-12; 15.7±4.4 pg/mL of TNF-α, as well as the chemokine MCP-1∶5.4±6.0 ng/mL (Fig 5A–E). Non-activated *L. amazonensis*-infected macrophages secreted mediators at levels similar to those produced by control cells: 49.1±3.7 pg/mL of IL-10; 17.7±1.7 pg/mL of IL-12; 17.9±2.2 pg/mL of TNF-α and 6.2±0.8 ng/mL of MCP-1, except for IL-6 which increased production to 16.7±3.0 pg/mL (Fig 5A–E). By contrast, non-activated infected macrophage cultures treated with 17-AAG exhibited a statistically significant reduction in the secretion of all produced cytokines: 8.3±0.3 pg/mL of IL-6; 40.8±2.0 pg/mL of IL-10; 11.4±0.7 pg/mL of TNF-α, as well as MCP-1∶0.6±0.3 ng/mL (**p<0.001; ***p<0.0001), with the exception of IL-12, which was released at 15.5±2.4 pg/ml, values similar to those produced by untreated and infected macrophages (*p* = 0.1) (Fig. 5A–E).

Uninfected macrophages that were activated, i.e., stimulated with IFN-γ, secreted elevated levels of mediators: 24.7±4.8 pg/mL of IL-12; 86.4±5.9 pg/mL of TNF-α, and 60.7 ng/mL of MCP-1, yet IL-6 and IL-10 production remained unaltered. When 17-AAG was added to both activated and infected macrophages, the following statistically significant reductions in secretion levels were observed in comparison to untreated cells: IL-6, from 13.0±2.3 (*p* = 0.0012) to 8.4±1; TNF-α, from 52.1±2.5 pg/mL (*p* = 0.0055) to 33.6±2.7 pg/mL; and MCP-1, from 12.2±8.4 ng/mL (*p* = 0.0084) to 0.9±0.2 ng/mL (Fig 5A,D–E). In sum, 17-AAG did not enhance macrophage production of either inflammatory cytokines or the MCP-1 chemokine, indicating that the mechanism involved in 17-AAG-induced parasite killing is unrelated to macrophage activation.

### Treatment with 17-AAG Induced Ultrastructural Alterations in Intracellular Parasites

**Figure 6:**
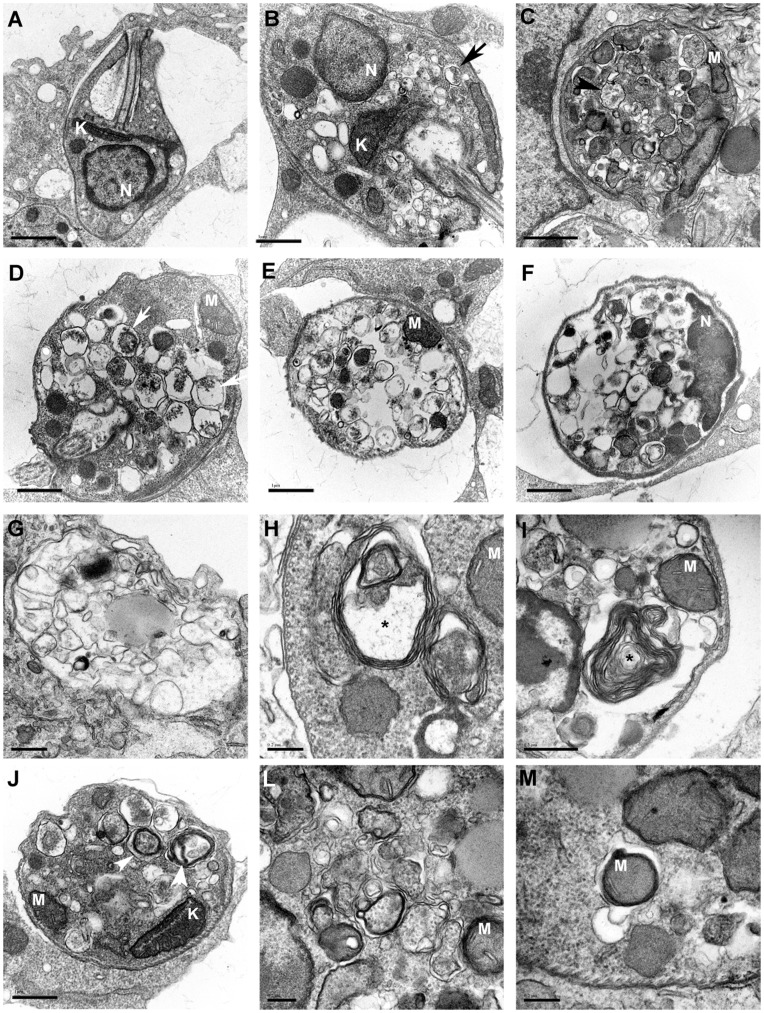
Alterations suggestive of autophagy in intracellular parasites treated with 17-AAG. Transmission electron microscopy was used to investigate ultrastructural morphological alterations in intracellular parasites inside 17-AAG-treated macrophages. (**A**) Control infected macrophages. After 12 h of treatment, several morphological alterations were seen in intracellular parasites, including: (**B**) numerous small vesicles some containing cytoplasmic material inside **(black arrow)**, (**C**) vacuoles larger in size **(black arrow-head)**, (**D**) with intravacuolar materials degraded **(white arrow)**. After 24 h of treatment, the intracellular parasites presented a large number of vesicles occupying most of the cytoplasm containing well-preserved nuclei, mitochondria, and subpellicular microtubules (**E–F**). After 48 h of treatment, no preserved parasites inside cells were observed, yet empty vesicles, and membrane-bounded structures with an electron-density similar to parasite cytosol in parasitophorous vacuoles were seen (**G**). At 12 and 24 h after treatment, several alterations were also visible in parasite cytoplasm, including myelin figures (*) (**H–I**), vesicles with double-layered membranes **(white arrow-head)** (**J–M**)**,** and portions of mitochondria inside membrane-bounded structures (**M**). The nuclei (**N**) and mitochondria (**M**) and kinetoplast (**K**) remained intact in all groups.

To further examine the mechanism involved in parasite killing, the authors used electron microscopy to investigate the presence of ultrastructural morphological alterations in intracellular parasites resulting from treatment with 17-AAG (500 nM). After 6 and 12 h of treatment, most intracellular parasites showed features similar to those found in control untreated macrophages (Fig. 6A). However, after 12 h of treatment, some intracellular parasites presented morphological ultrastructural alterations (Fig. 6). First, several small vesicles were observed in the cytoplasm of parasites, some even containing cytoplasmic material inside (Fig. 6B, arrows). It appeared as though the vacuoles had grown in size and that intravacuolar materials had been degraded (Fig. 6C–D). At 24 h after treatment, the intracellular parasites presented a large number of vesicles occupying most of the cytoplasm, even though preserved subpellicular microtubules, as well as intact nuclei and well-preserved mitochondria were observed (Fig. 6E–F). At 48 h, membrane-bounded structures were found inside parasitophorous vacuoles, probably corresponding to the remains of dead parasites (Fig 6G). By contrast, most of the untreated cells contained well-preserved round amastigotes within parasitophorous vacuoles (data not shown). At 12 and 24 h after treatment, several alterations suggestive of autophagy were also visible in the cytoplasm of parasites, including myelin figures (Fig. 6H–I), vesicles with double-layered membranes (Fig. 6J–L) and portions of mitochondria inside membrane-bounded structures (Fig. 6M).

In order to assess the possibility of autophagy being involved in parasite death, infected macrophages were simultaneously treated with 17-AAG in addition to wortmannin (300 nM), which inhibits autophagy by a mechanism dependent on phosphatidylinositol 3-kinase (PI3K). After 48 h of infection, the number of viable parasites was assessed by replacing culture medium with Schneider’s complete medium at the end of incubation period. In cells treated with 17-AAG (500 nM) and wortmannin (300 nM), the number of viable parasites (706.7±126.9 parasites/mL) was two times higher than in macrophages treated with 17-AAG alone (345±154.4 parasites/mL). Nonetheless, no statistically significant difference was observed between these groups (*p* = 0.08). We observed the same result when *Leishmania* were pre-treated with wortmannin and when different concentrations of 17-AAG were used (25 and 125 nM), suggesting that 17-AAG induced parasite death by way of a mechanism independent of PI3K.

## Discussion

Protozoan parasites undergo drastic environmental changes throughout their development process, and evidence indicates that HSP90 is a fundamental molecular chaperone throughout the life cycle of a variety of protozoa, including *Trypanosoma cruzi*, *Toxoplasma gondii* and *Plasmodium falciparum* [34]. It follows, then, that HSP90 may be a candidate target for the chemotherapeutic treatment of parasitic diseases [11], [18], [21], [25], [34]. The present study represents the first attempt to investigate the effects of an HSP90 inhibitor, namely 17-AAG, on axenic cultures of *L. amazonensis* promastigotes, as well as on *L. amazonensis* parasites within infected macrophages.

We demonstrated that the number of axenic *L. amazonensis* promastigotes remained stable in a time- and dose-dependent manner after cultures were treated with 17-AAG (Fig. 1A). Additionally, the promastigotes treated with this inhibitor became rounded in shape and lost flagellum (data not shown). A previous study demonstrated that the growth of *L. donovani* was inhibited when axenic cultures were treated with GA, a 17-AAG analog, due to parasite growth arrest in the G2 cell cycle phase [18]. In this previous study, treatment with GA further induced the transformation of *L. donovani* promastigotes into rounded amastigote-like forms, alterations similar to those observed in promastigotes submitted to conditions mimicking the parasitophorous vacuole microenvironment, where these parasites transform and multiply in their amastigote forms. These findings, taken together with those of the present study, clearly demonstrate that HSP90 inhibitors directly induce a cytostatic effect on axenic cultures of *Leishmania* spp. promastigotes, as well as promote the transformation of promastigote forms into amastigote-like forms [18].

In the present study, treatment with 17-AAG drastically reduced the intracellular viability of *L. amazonensis* (Fig. 3). Similarly, previous studies demonstrated that a GA-induced inhibition of HSP90 blocked the growth of other protozoa, such as *P. falciparum,* within erythrocytes both *in vitro* [19], [20], [35] and in an *in vivo* experimental malaria model [21]. These findings reinforce the notion that parasite HSP90 is essential to intracellular parasite growth and complete differentiation process [36]. The present study detected an inhibitory effect from 17-AAG even when administered at low concentrations of 125 to 500 nM, under brief exposure periods and in late stages of infection. This inhibitory action, associated to a low SI value, suggest that 17-AAG may be appropriate for use in *in vivo* studies, which could prove advantageous.

Furthermore, there is evidence that 17-AAG is liposoluble and that this inhibitor accumulates intracellularly in cancer cells [37]. Although the present study did not seek to address the intracellular distribution of 17-AAG in infected cells, this inhibitor may have an enhanced intracellular effect, however this remains unverified. It is possible that 17-AAG accumulates in *Leishmania*-induced parasitophorous vacuoles, which are membrane-bound compartments with lysosomal characteristics, an acidic pH and abundant hydrolytic enzymes [38], [39]. This environment is ideal for the destruction of parasites subjected to the action of both microbicidal molecules and antileishmanial drugs.

Alternatively, it is also possible that 17-AAG acts on host HSP90 instead of directly affecting parasite HSP90, thereby promoting the production of molecules known for microbicidal action, including O_2_^−^ and NO [40], as well as pro-inflammatory mediators [40], [41]. However, the present study demonstrated that infected macrophages treated with 17-AAG produced reduced levels of O_2_^−^ and NO (Fig. 4), as well as pro-inflammatory mediators, including TNF-α, IL-6 and MCP-1 (Fig. 5). Additionally, previous studies demonstrated that HSP90 inhibitors similarly induced anti-oxidative [42], [43], [44], [45] and anti-inflammatory responses in *in vivo* and *in vitro* infection models not involving *Leishmania* [46], [47], [48]. Taken together, these findings directly contradict the literature, which clearly evidences the production of proinflammatory mediators TNF-α, IL-1β, IL-6, IL-12, IFN-γ and MCP-1 being associated with the control of *Leishmania* spp infection [40], [49], [50], [51]. To the best of our knowledge, this is the first report of treatment with an HSP-90 inhibitor being associated with low levels of pro-inflammatory molecule production by *Leishmania*-infected macrophages in association with the clearance of intracellular parasites. Therefore, the findings presented herein indicate that 17-AAG-induced *L. amazonensis* killing in the absence of host pro-inflammatory molecule production, thereby contradicting the known role these molecules play in control of parasite infection. This reinforces the notion that 17-AAG directly affects parasite survival while overriding the drug’s potential toxicity with respect to host cells.

To investigate the mechanism of parasite killing at the ultrastructural level, the authors employed transmission electron microscopy. Images revealed alterations in intracellular parasites suggestive of autophagy (Fig. 6). Autophagy is a naturally occurring process in *Leishmania* infection, which plays an important role in the differentiation process from promastigote to amastigote [52], [53]. The present study showed that infected macrophages treated with wortmannin, a PI3K inhibitor known to disrupt the autophagic process [52], were unable to reverse induced parasite death following treatment with 17-AAG. The inability of wortmannin to revert the autophagic process may be related to the fact that 17-AAG irreversibly affected parasite viability at early stages of treatment. Furthermore, the lack of reversibility in the autophagic process may be related to the possibility that wortmannin is unable to access the PI3K target molecule present in parasites within parasitophorous vacuoles. Since the autophagic process leads to the release of energy and is triggered in cells subjected to stress conditions [54], it is possible that the parasite death observed herein did not result from the onset of autophagy, but rather that the autophagic-like process was related to *Leishmania*’s attempt to evade the action of 17-AAG. The absence of nuclear or mitochondrial changes in the parasites themselves leads us to suggest that apoptosis played no role in this particular mechanism of parasite death. Nonetheless, the underlying mechanism involved in 17-AAG-induced parasite death remains to be clarified.

In sum, the authors propose that the treatment of cutaneous leishmaniasis with 17-AAG may represent a promising therapeutic strategy for the elimination of intracellular *Leishmania*, as well as offering the added benefits of reduced levels of O_2_^−^ and NO, in addition to lower proinflammatory mediator production. This treatment method would be especially advantageous in lesions typical of cutaneous and muco-cutaneous leishmaniasis, which are characterized by an intense inflammatory response, low parasite counts, elevated expressions of IFN-γ and TNF-α, as well as pronounced tissue damage as a result of intensified oxidative molecule production [55], [56], [57]. Although 17-AAG is currently in clinical trials for the treatment of neoplasia [58], [59], [60], this medication has yet to receive approval for human treatment despite many advances made in recent years with respect to its formulation in an effort to reduce toxicity and increase the threshold dosage [58]. It is our hope that these trials, in conjunction with the findings presented herein, will provide a basis for this inhibitor’s eventual application in the treatment of patients suffering from leishmaniasis.

## References

[1] Desjeux P (2004) Leishmaniasis: current situation and new perspectives. Comp Immunol Microbiol Infect Dis 27: 305–318. 15225981 10.1016/j.cimid.2004.03.004

[2] Kedzierski L, Zhu Y, Handman E (2006) *Leishmania* vaccines: progress and problems. Parasitology 133 Suppl: S87–112 10.1017/S0031182006001831 17274851

[3] Croft SL, Coombs GH (2003) Leishmaniasis–current chemotherapy and recent advances in the search for novel drugs. Trends Parasitol 19: 502–508. 14580961 10.1016/j.pt.2003.09.008

[4] Tiuman TS, Santos AO, Ueda-Nakamura T, Filho BP, Nakamura CV (2011) Recent advances in leishmaniasis treatment. Int J Infect Dis 15: e525–532. 21605997 10.1016/j.ijid.2011.03.021

[5] Croft SL, Sundar S, Fairlamb AH (2006) Drug resistance in leishmaniasis. Clin Microbiol Rev 19: 111–126. 16418526 10.1128/CMR.19.1.111-126.2006 PMC1360270

[6] Antoniou T, Gough KA (2005) Early-onset pentamidine-associated second-degree heart block and sinus bradycardia: case report and review of the literature. Pharmacotherapy 25: 899–903. 15927910 10.1592/phco.2005.25.6.899

[7] Assan R, Perronne C, Assan D, Chotard L, Mayaud C, et al (1995) Pentamidine-induced derangements of glucose homeostasis. Determinant roles of renal failure and drug accumulation. A study of 128 patients. Diabetes Care 18: 47–55. 7698047 10.2337/diacare.18.1.47

[8] Chappuis F, Sundar S, Hailu A, Ghalib H, Rijal S, et al (2007) Visceral leishmaniasis: what are the needs for diagnosis, treatment and control? Nat Rev Microbiol 5: 873–882. 17938629 10.1038/nrmicro1748

[9] Seifert K (2011) Structures, targets and recent approaches in anti-leishmanial drug discovery and development. Open Med Chem J 5: 31–39. 21629509 10.2174/1874104501105010031 PMC3103891

[10] Grenert JP, Sullivan WP, Fadden P, Haystead TA, Clark J, et al (1997) The amino-terminal domain of heat shock protein 90 (HSP90) that binds geldanamycin is an ATP/ADP switch domain that regulates Hsp90 conformation. J Biol Chem 272: 23843–23850. 9295332 10.1074/jbc.272.38.23843

[11] Neckers L (2002) Hsp90 inhibitors as novel cancer chemotherapeutic agents. Trends Mol Med 8: S55–61. 11927289 10.1016/s1471-4914(02)02316-x

[12] Prodromou C, Roe SM, O’Brien R, Ladbury JE, Piper PW, et al (1997) Identification and structural characterization of the ATP/ADP-binding site in the Hsp90 molecular chaperone. Cell 90: 65–75. 9230303 10.1016/s0092-8674(00)80314-1

[13] Gomez-Monterrey I, Sala M, Musella S, Campiglia P (2012) Heat Shock Protein 90 Inhibitors as Therapeutic Agents. Recent Pat Anticancer Drug Discov. 10.2174/157489212801820066 22338602

[14] Whitesell L, Lindquist SL (2005) HSP90 and the chaperoning of cancer. Nat Rev Cancer 5: 761–772. 16175177 10.1038/nrc1716

[15] Folgueira C, Requena JM (2007) A postgenomic view of the heat shock proteins in kinetoplastids. FEMS Microbiol Rev 31: 359–377. 17459115 10.1111/j.1574-6976.2007.00069.x

[16] Nathan DF, Vos MH, Lindquist S (1997) *In vivo* functions of the *Saccharomyces cerevisiae* Hsp90 chaperone. Proc Natl Acad Sci U S A 94: 12949–12956. 9371781 10.1073/pnas.94.24.12949 PMC24244

[17] Scheibel T, Buchner J (1998) The Hsp90 complex–a super-chaperone machine as a novel drug target. Biochem Pharmacol 56: 675–682. 9751071 10.1016/s0006-2952(98)00120-8

[18] Wiesgigl M, Clos J (2001) Heat shock protein 90 homeostasis controls stage differentiation in *Leishmania donovani* . Mol Biol Cell 12: 3307–3316. 11694568 10.1091/mbc.12.11.3307 PMC60256

[19] Banumathy G, Singh V, Pavithra SR, Tatu U (2003) Heat shock protein 90 function is essential for *Plasmodium falciparum* growth in human erythrocytes. J Biol Chem 278: 18336–18345. 12584193 10.1074/jbc.M211309200

[20] Kumar R, Musiyenko A, Barik S (2005) Plasmodium falciparum calcineurin and its association with heat shock protein 90: mechanisms for the antimalarial activity of cyclosporin A and synergism with geldanamycin. Mol Biochem Parasitol 141: 29–37. 15811524 10.1016/j.molbiopara.2005.01.012

[21] Pallavi R, Roy N, Nageshan RK, Talukdar P, Pavithra SR, et al (2010) Heat shock protein 90 as a drug target against protozoan infections: biochemical characterization of HSP90 from *Plasmodium falciparum* and *Trypanosoma evansi* and evaluation of its inhibitor as a candidate drug. J Biol Chem 285: 37964–37975. 20837488 10.1074/jbc.M110.155317 PMC2992230

[22] Ahn HJ, Kim S, Nam HW (2003) Molecular cloning of the 82-kDa heat shock protein (HSP90) of *Toxoplasma gondii* associated with the entry into and growth in host cells. Biochem Biophys Res Commun 311: 654–659. 14623321 10.1016/j.bbrc.2003.10.045

[23] Brandau S, Dresel A, Clos J (1995) High constitutive levels of heat-shock proteins in human-pathogenic parasites of the genus *Leishmania* . Biochem J 310 (Pt 1): 225–232. 10.1042/bj3100225 PMC1135877 7646449

[24] Li Q, Zhou Y, Yao C, Ma X, Wang L, et al (2009) Apoptosis caused by Hsp90 inhibitor geldanamycin in *Leishmania donovani* during promastigote-to-amastigote transformation stage. Parasitol Res 105: 1539–1548. 19690889 10.1007/s00436-009-1582-y

[25] Shonhai A, Maier AG, Przyborski JM, Blatch GL (2011) Intracellular protozoan parasites of humans: the role of molecular chaperones in development and pathogenesis. Protein Pept Lett 18: 143–157. 20955165 10.2174/092986611794475002

[26] Schulte TW, Neckers LM (1998) The benzoquinone ansamycin 17-allylamino-17-demethoxygeldanamycin binds to HSP90 and shares important biologic activities with geldanamycin. Cancer Chemother Pharmacol 42: 273–279. 9744771 10.1007/s002800050817

[27] Isaacs JS, Xu W, Neckers L (2003) Heat shock protein 90 as a molecular target for cancer therapeutics. Cancer Cell 3: 213–217. 12676580 10.1016/s1535-6108(03)00029-1

[28] Gomes IN, Calabrich AF, Tavares Rda S, Wietzerbin J, de Freitas LA, et al (2003) Differential properties of CBA/J mononuclear phagocytes recovered from an inflammatory site and probed with two different species of *Leishmania* . Microbes Infect 5: 251–260. 12706438 10.1016/s1286-4579(03)00025-x

[29] Veras PS, Welby-Borges M, de Santana CD, Nihei J, Cardillo F, et al (2006) *Leishmania amazonensis*: participation of regulatory T and B cells in the in vitro priming (PIV) of CBA/J spleen cells susceptible response. Exp Parasitol 113: 201–205. 16516200 10.1016/j.exppara.2006.01.008

[30] Hothersall JS, Gordge M, Noronha-Dutra AA (1998) Inhibition of NADPH supply by 6-aminonicotinamide: effect on glutathione, nitric oxide and superoxide in J774 cells. FEBS Lett 434: 97–100. 9738459 10.1016/s0014-5793(98)00959-4

[31] Ding AH, Nathan CF, Stuehr DJ (1988) Release of reactive nitrogen intermediates and reactive oxygen intermediates from mouse peritoneal macrophages. Comparison of activating cytokines and evidence for independent production. J Immunol 141: 2407–2412. 3139757

[32] de Sa MS, Costa JF, Krettli AU, Zalis MG, Maia GL, et al (2009) Antimalarial activity of betulinic acid and derivatives in vitro against *Plasmodium falciparum* and *in vivo* in *P. berghei*-infected mice. Parasitol Res 105: 275–279. 19367418 10.1007/s00436-009-1394-0

[33] Courret N, Frehel C, Prina E, Lang T, Antoine JC (2001) Kinetics of the intracellular differentiation of *Leishmania amazonensis* and internalization of host MHC molecules by the intermediate parasite stages. Parasitology 122: 263–279. 11289063 10.1017/s0031182001007387

[34] Roy N, Nageshan RK, Ranade S, Tatu U (2012) Heat shock protein 90 from neglected protozoan parasites. Biochim Biophys Acta 1823: 707–711. 22198098 10.1016/j.bbamcr.2011.12.003

[35] Kumar R, Musiyenko A, Barik S (2003) The heat shock protein 90 of *Plasmodium falciparum* and antimalarial activity of its inhibitor, geldanamycin. Malar J 2: 30. 14514358 10.1186/1475-2875-2-30 PMC201030

[36] Neckers L, Tatu U (2008) Molecular chaperones in pathogen virulence: emerging new targets for therapy. Cell Host Microbe 4: 519–527. 19064253 10.1016/j.chom.2008.10.011 PMC2752846

[37] Chiosis G, Huezo H, Rosen N, Mimnaugh E, Whitesell L, et al (2003) 17AAG: low target binding affinity and potent cell activity–finding an explanation. Mol Cancer Ther 2: 123–129. 12589029

[38] Antoine JC, Prina E, Jouanne C, Bongrand P (1990) Parasitophorous vacuoles of *Leishmania amazonensis*-infected macrophages maintain an acidic pH. Infect Immun 58: 779–787. 1689700 10.1128/iai.58.3.779-787.1990 PMC258533

[39] Prina E, Antoine JC, Wiederanders B, Kirschke H (1990) Localization and activity of various lysosomal proteases in *Leishmania amazonensis*-infected macrophages. Infect Immun 58: 1730–1737. 2187806 10.1128/iai.58.6.1730-1737.1990 PMC258715

[40] Liese J, Schleicher U, Bogdan C (2008) The innate immune response against *Leishmania* parasites. Immunobiology 213: 377–387. 18406382 10.1016/j.imbio.2007.12.005

[41] Sacks D, Noben-Trauth N (2002) The immunology of susceptibility and resistance to *Leishmania major* in mice. Nat Rev Immunol 2: 845–858. 12415308 10.1038/nri933

[42] Chen F, Pandey D, Chadli A, Catravas JD, Chen T, et al (2011) Hsp90 regulates NADPH oxidase activity and is necessary for superoxide but not hydrogen peroxide production. Antioxid Redox Signal 14: 2107–2119. 21194376 10.1089/ars.2010.3669 PMC3085945

[43] Kone BC, Kuncewicz T, Zhang W, Yu ZY (2003) Protein interactions with nitric oxide synthases: controlling the right time, the right place, and the right amount of nitric oxide. Am J Physiol Renal Physiol 285: F178–190. 12842859 10.1152/ajprenal.00048.2003

[44] Luo S, Wang T, Qin H, Lei H, Xia Y (2011) Obligatory role of heat shock protein 90 in iNOS induction. Am J Physiol Cell Physiol 301: C227–233. 21430289 10.1152/ajpcell.00493.2010 PMC3129818

[45] Yoshida M, Xia Y (2003) Heat shock protein 90 as an endogenous protein enhancer of inducible nitric-oxide synthase. J Biol Chem 278: 36953–36958. 12855682 10.1074/jbc.M305214200

[46] Chatterjee A, Dimitropoulou C, Drakopanayiotakis F, Antonova G, Snead C, et al (2007) Heat shock protein 90 inhibitors prolong survival, attenuate inflammation, and reduce lung injury in murine sepsis. Am J Respir Crit Care Med 176: 667–675. 17615388 10.1164/rccm.200702-291OC PMC1994236

[47] Poulaki V, Iliaki E, Mitsiades N, Mitsiades CS, Paulus YN, et al (2007) Inhibition of Hsp90 attenuates inflammation in endotoxin-induced uveitis. FASEB J 21: 2113–2123. 17400913 10.1096/fj.06-7637com

[48] Wax S, Piecyk M, Maritim B, Anderson P (2003) Geldanamycin inhibits the production of inflammatory cytokines in activated macrophages by reducing the stability and translation of cytokine transcripts. Arthritis Rheum 48: 541–550. 12571865 10.1002/art.10780

[49] Moll H (1997) The role of chemokines and accessory cells in the immunoregulation of cutaneous leishmaniasis. Behring Inst Mitt: 73–78. 9303204

[50] Titus RG, Sherry B, Cerami A (1989) Tumor necrosis factor plays a protective role in experimental murine cutaneous leishmaniasis. J Exp Med 170: 2097–2104. 2584936 10.1084/jem.170.6.2097 PMC2189541

[51] Romagnani S (1991) Type 1 T helper and type 2 T helper cells: functions, regulation and role in protection and disease. Int J Clin Lab Res 21: 152–158. 1687725 10.1007/BF02591635

[52] Besteiro S, Williams RA, Morrison LS, Coombs GH, Mottram JC (2006) Endosome sorting and autophagy are essential for differentiation and virulence of *Leishmania major* . J Biol Chem 281: 11384–11396. 16497676 10.1074/jbc.M512307200

[53] Williams RA, Tetley L, Mottram JC, Coombs GH (2006) Cysteine peptidases CPA and CPB are vital for autophagy and differentiation in *Leishmania mexicana* . Mol Microbiol 61: 655–674. 16803590 10.1111/j.1365-2958.2006.05274.x

[54] Shintani T, Klionsky DJ (2004) Autophagy in health and disease: a double-edged sword. Science 306: 990–995. 15528435 10.1126/science.1099993 PMC1705980

[55] Lessa HA, Machado P, Lima F, Cruz AA, Bacellar O, et al (2001) Successful treatment of refractory mucosal leishmaniasis with pentoxifylline plus antimony. Am J Trop Med Hyg 65: 87–89. 11508396 10.4269/ajtmh.2001.65.87

[56] Miller RA, Britigan BE (1997) Role of oxidants in microbial pathophysiology. Clin Microbiol Rev 10: 1–18. 8993856 10.1128/cmr.10.1.1 PMC172912

[57] Oliveira F, Bafica A, Rosato AB, Favali CB, Costa JM, et al (2011) Lesion size correlates with *Leishmania* antigen-stimulated TNF-α levels in human cutaneous leishmaniasis. Am J Trop Med Hyg 85: 70–73. 21734128 10.4269/ajtmh.2011.10-0680 PMC3122347

[58] Kim YS, Alarcon SV, Lee S, Lee MJ, Giaccone G, et al (2009) Update on Hsp90 inhibitors in clinical trial. Curr Top Med Chem 9: 1479–1492. 19860730 10.2174/156802609789895728 PMC7241864

[59] Neckers L, Workman P (2012) Hsp90 molecular chaperone inhibitors: are we there yet? Clin Cancer Res 18: 64–76. 22215907 10.1158/1078-0432.CCR-11-1000 PMC3252205

[60] Usmani SZ, Bona R, Li Z (2009) 17 AAG for HSP90 inhibition in cancer–from bench to bedside. Curr Mol Med 9: 654–664. 19601813 10.2174/156652409788488757

